# Health-related quality of life and its determinants in children with a congenital diaphragmatic hernia

**DOI:** 10.1186/1750-1172-8-89

**Published:** 2013-06-20

**Authors:** Fabrice Michel, Karine Baumstarck, Agathe Gosselin, Pierre Le Coz, Thierry Merrot, Sophie Hassid, Kathia Chaumoître, Julie Berbis, Claude Martin, Pascal Auquier

**Affiliations:** 1 Espace Ethique Méditerranéen, ADES UMR 7268 ADES, Espace Ethique Méditerranéen Aix-Marseille Université, Marseille 13385, France; 2 Pediatric and Neonatal Intensive Care Unit, North Hospital, APHM, Marseille 13915, France; 3 EA3279, Self-perceived Health Assessment Research Unit, School of Medicine, Aix-Marseille Université, Marseille F-13385, France; 4 Pediatric Surgery Unit, North Hospital Nord, APHM, Aix-Marseille Université, Marseille 13915, France; 5 Department of Medical Imaging, North Hospital, AP-HM, Marseille 13915, France

**Keywords:** Congenital diaphragmatic hernia, Survivors, Quality of life, Kidscreen-27, SF36

## Abstract

**Background:**

The development of new therapeutics has led to progress in the early management of congenital diaphragmatic hernia (CDH) in pediatric intensive care units (PICU). Little is known about the impact on the quality of life (QoL) of children and their family. The aim of this study was to assess the impact of CDH treated according to the most recent concepts and methods outlined above on child survivors’ QoL and their parents’ QoL.

**Patients and methods:**

This study incorporated a cross-sectional design performed in two PICU (Marseille, France). Families of CDH survivors born between 1999 and 2008 were eligible. The following data were recorded: socio-demographics, antenatal history and delivery, initial hospitalization history. Self-reported data were collected by mail, including current clinical problems of the children (13-symptom list), children’s QoL (Kidscreen-27 questionnaire), and parents’ QoL (Short-Form 36 questionnaire). Children’s QoL score was compared with controls and QoL of survivors of childhood leukemia. Parent’s QoL was compared with controls. Non-parametric statistics were employed.

**Results:**

Forty-two families agreed to participate and questionnaires were completed by 32 of them. Twenty-one children had a current clinical problems related to CDH. All the QoL scores of CHD survivors were significantly lower compared with controls. The physical well-being dimension was significantly higher for CHD survivors compared with survivors of childhood leukemia. Gastro-esophageal reflux at discharge, antenatal diagnosis, length of stay in the PICU, and neuropsychological and respiratory issues significantly impacted QoL scores of children. The parents of CHD survivors had significantly poorer score in emotional role dimension compared with controls.

**Conclusion:**

The impact of CDH on QoL seems to be important and must be understood by clinicians who treat these children and their parents.

## Background

Congenital diaphragmatic hernia (CDH) is a rare but severe congenital malformation [1]. Advances in prenatal imaging have allowed for better estimating the severity and prognosis before birth according to criteria evaluating lung volume. Additionally, a better understanding of pathophysiology and the development of new therapeutics has led to progress in the early management of CDH in pediatric intensive care units [2]. Since 1990, stabilization before surgery, protective mechanical ventilation, permissive hypercapnia, high-frequency oscillation, inhaled nitric oxide (INO) and other vasodilator drugs for pulmonary hypertension have been developed [3] and are the clinician’s common weapons [4]. When extracorporeal membrane oxygenation (ECMO) is available, some units can use it as rescue therapy. Despite this progress, the mortality rate reported by most of the units that tend to these patients remains between 30 and 50%, reflecting the severity of the disease [5]. Furthermore, recent studies showed that persistent pulmonary hypertension, pulmonary hypoplasia, and non-life-threatening associated malformations are responsible for important morbidity in survivors [6]. The most frequent disorders are pulmonary issues associated with dyspnea, bronchospasm, chronic lung disease, prolonged oxygen delivery and/or tracheotomy [7], gastrointestinal issues, including a particularly high incidence of gastroesophageal reflux (GER) disease [8], and neurocognitive and behavioral disorders in some serious cases [9]. Psychological disorders, growth failure, hearing loss and musculoskeletal abnormalities are also frequent in survivors [6,8,10,11]. Although all these issues have been widely reported, little is known about their impact on the quality of life (QoL) of children and their family.

To our knowledge, only 4 previous studies have specifically explored the QoL of CDH survivors. One concerned only adults [12], and 3 explored the QoL of children or their parents [13-15]. While adult survivors have a similar QoL as other adults, the studies focusing on children’s QoL have revealed significantly lower QoL level compared to controls [13] and norms [15]. A study that evaluated the impact on family and parental well-being [14] showed that the presence of medical issues in children was associated with a greater impairment of family functioning. No authors have explored QoL of a homogeneous group of CDH survivors treated according to the most recent therapeutic management [4]. These new concepts are probably associated with better outcome, with particularly less respiratory sequelae. And the other hand, possibly, higher survival rate of children with severe disease, can lead to higher risk of major respiratory, neurologic, digestive, orthopedic, psychological or others sequelae impacting QoL.

The main objective of our study was to assess the impact of CDH treated according to the most recent concepts and methods outlined above on child survivors’ QoL and their parents’ QoL. The secondary objectives were first, to compare QoL levels of the CDH survivors with those observed in the general population and another pathology associated with long-term consequences (childhood leukemia survivors), and second, to determine the impact of clinical and socio-demographic factors on children’s and parents’ QoL scores.

## Methods

### Study design and population

This study incorporated a cross-sectional design and was performed in two pediatric intensive care units of a French public academic teaching hospital (Marseille, France). The inclusion criteria were as follows: children with a diagnosis of CDH born between January 1997 and December 2008 who were initially treated according to concepts and methods developed during the nineties (i.e., protective mechanical ventilation, including permissive hypercapnia and preoperative stabilization, INO availability) and parents or legal guardians authorizing participation in the study and child consent if older than 6. When the diagnosis of CDH was made after discharge of maternity the patient was excluded. According to French law, the Consultative Committee for the data processing in health research (CCTIRS) approved this research, and no more authorizations were requested for this study. The medical database allowed for identifying the eligible children according to the selection criteria. Mail was sent to the parents describing the objectives of the study. In cases of participation acceptance, the parents returned written consent. Questionnaires were subsequently sent by mail.

### Medical records

The following data were collected from the medical records: 1. sociodemographic: age and gender of the child; 2. antenatal history and delivery: prenatal diagnosis (yes/no), birth term, birth weight, and caesarean (yes/no); and 3. initial hospitalization history: CDH side (left/right), date and nature of the initial surgery (simple/not simple, *ie*, requiring progressive closure and/or requiring a patch), initial administration (yes/no) and administration duration of surfactant, amines, and INO, mechanical ventilation duration, sedation duration, need for ECMO and duration, initial pediatric intensive care unit (PICU) and hospitalization duration, weight at discharge, and GER at discharge.

### Self-reported data

Self-reported data were collected from the children themselves and their parents. The following self-reported data were collected from the parents:

1. Current staturo-ponderal information: weight and body mass index Z-score.

2. Current clinical problems caused by CDH: the parents were questioned about the frequency (never or sometimes/frequent or very frequent) of current clinical problems using a checklist exploring 13 symptoms in 4 main clinical domains: digestive symptoms (vomiting, reflux, retrosternal burning, constipation, diarrhea, and abdominal pain), respiratory symptoms (dyspnea at rest, exertional dyspnea, nocturnal dyspnea, chest pain, repeated respiratory tract infection, hospitalization for respiratory issue, and long-term respiratory treatment), neuropsychological symptoms (cerebral palsy, mental retardation, and neuropsychological disorders) or orthopedic symptoms (difficulty walking, physiotherapy, and orthopedic treatment). The content of the checklist was defined in accordance with the literature and was considered relevant by 3 members of the study group (a pediatrician, a pediatric surgeon, and a psychologist).

3. Behavior of the child: a brief behavioral screening was performed using the Strengths and Difficulties Questionnaire (SDQ) [16]. The SDQ is a 25-item questionnaire for approximately 3- to 16-year-old children exploring 5 dimensions: emotional symptoms (5 items), conduct problems (5), hyperactivity/inattention (5), peer relationship problems (5), and prosocial behavior (5). Higher scores indicate a higher level of difficulties.

### Evaluation of the quality of life

The QoL of the children was assessed using the Kidscreen-27 questionnaire [17], which is a well-validated generic 27-item questionnaire describing 5 dimensions: physical well-being (5 items), psychological well-being (7 items), parents and autonomy (4 items), peers and social support (4 items), and school (4 items). Two versions were available: a parent version for children under 8 years (the parent assesses the QoL of the child) and a child version for children aged over 8 years (the child completes the questionnaire). The level of agreement between children and their parents was previously assessed [18]. French norms are available (http://www.kidscreen.org) [19,20].

The QoL of the parents was also assessed using the Short-Form 36 (SF36) questionnaire, which is a generic questionnaire used worldwide [21] describing eight subscales (physical function, social functioning, role physical, role emotional, mental health, vitality, bodily pain, and general health). Two composite scores (physical and mental) can also be calculated. French norms are available [22].

The scores of the 3 questionnaires ranged between 0 and 100, with higher scores indicating a better QoL.

### Statistical analysis

Continuous variables were expressed as the means and standard deviations and medians and ranges. Qualitative variables were expressed as percentages. Non-parametric statistics were employed. The Kidscreen scores of children were compared to those obtained from French age- and sex-matched controls from a normal sample of 1000 subjects [23] and a sample of childhood leukemia survivors of the “L.E.A” cohort (Leucémie de l’Enfant et de l’Adolescent – childhood and adolescent leukemia), a French multi-center historical and prospective cohort of prevalent and incident cases from 2004–2009 [24]. The SF36 scores [21] of parents were compared to those obtained from French age- and sex-matched controls from a normal sample of 3656 subjects [22]. Comparisons of mean QoL scores between different sub-groups (current clinical problems, gender of the child, CDH side, antenatal diagnosis, and GER at discharge) were performed using Mann–Whitney tests. Associations between QoL scores and continuous variables (age, PICU, and LOS) were analyzed using Spearman’s correlation tests. The statistical analyses were performed using SPSS software package version 17.0 (SPSS Inc., Chicago, IL, USA). All tests were two sided. Statistical significance was defined as p < 0.05.

## Results

### Population

Ninety-nine children were treated for CDH in the two PICUs during the period. Fifty-seven (57.5%) were discharged alive. Five of them were excluded from analysis because the diagnosis was made after maternity discharge. Ten of the 52 remaining families were not found. The 42 other families agreed to participate in the study, and parents and children given their informed consent. Questionnaires were completed by 32 (76.2%) of the 42 families. Among them, only one has been treated with ECMO. At the time of observation, 8 (25.0%) children were less than 4 years old (2.5 ± 0.5 years), 14 (44%) were between 4 and 7 years old (5.1 ± 1.2 years), and 10 (31%) were 8 or older (10.4 ± 1.6 years). The mean weight of the children was 22.2 ± 8.9 Kg, and the BMI Z-score was −1.4 ± 1.9. The characteristics of the population are reported in Table 1.

**Table 1:** Patients characteristics

**1. Characteristics at the time of response**		**N = 32**
Gender	Girls n (%)	11 (34.4)
	Boys n (%)	21 (65.6)
Age (yrs)	M±SD	6.7 ± 3.3
	m (min/max)	5.8 (2.6/13.8)
Current BMI z-score	M±SD	−1.4 ± 1.5
	m (min/max)	−2,1(−4,0/2,2)
CDH side	Left n (%)	27 (84.4)
	Right n (%)	5 (15.6)
**2. Antenatal and neonatal data**		
Prenatal diagnosis	Yes n (%)	19 (59.4)
Birth weight (g)	M±SD	2996 ± 709
	m (min/max)	3100 (900/4000)
Term (WOG)	M±SD	38.2 ± 2.9
	m (min/max)	39 (27/41)
Birth weight z-score	M±SD	−0.5 ± 0.7
	m (min/max)	0.6(−2.2/1.2)
Caesarean	Yes n (%)	10 (31.3)
Surfactant	Yes n (%)	13 (40.6)
MV Duration (d)	M±SD	13.0 ± 12.2
	m (min/max)	10(1/57)
HFO	Yes n (%)	24 (75.0)
Duration (d)	M±SD	13.1 ± 13.2
	m (min/max)	9 (1/57)
Amines	Yes n (%)	23 (71.9)
Duration (d)	M±SD	7.4 ± 5.2
	m (min/max)	6 (1–26)
NO	Yes n (%)	19 (59.4)
Duration (d)	M±SD	6.9 ± 7.0
	m (min/max)	4 (1–25)
Sedation (d)	M±SD	10.7 ± 12.5
	m (min-max)	6 (1/57)
ICU LOS (d)	M±SD	18.6 ± 18.3
	m (min/max)	14 (3/92)
CDH repair (day of life)	M±SD	2.7 ± 2.3
	m (min/max)	1(1/12)
Surgery	Simple	27 (84.4)
	Others	5 (15.6)
Hospital LOS (d)	M±SD	40.2 ± 34.0
	m (min/max)	28(10/140)
Weight at discharge (g)	M±SD	3569 ± 829
	m (min/max)	3380 (1545/4855)
GER at discharge	Yes n(%)	14 (43.8)

*WOG* week of gestation, *LOS* length of stay, *BMI* Body mass index, *MV*, mechanical Ventilation, *HFO* High frequency oscillation, *NO* nitric oxide, *GER* Gastro-esophageal reflux.

*M±SD* mean±standard deviation, *m (min/max)*, median (minimum/maximum).

### Initial management and current clinical problems

The prenatal diagnosis of CDH was made by ultrasound in 19 (59%) cases at a mean term of 29.0 ± 3.9 weeks of gestation (WOG). Twelve children born after 2003 underwent antenatal magnetic resonance imaging. The mean ratio of total/expected pulmonary volume (TPV/EPV) was 74.5 ± 15.6%. The mean term was 38.2 ± 2.8 WOG, and the mean birth weight was 2996 ± 706 g. Data on birth and PICU management are reported in Table 1. Ten (31.3%) neonates were born by cesarean. Four children had associated malformations: three had upper urinary tract dilatation and one had a T5 hemivertebra. Twenty-four (75.0%) children were treated by high-frequency oscillation, 23 (71.9%) had aminergic treatment, and 19 (59.4) had INO. The only treatment with ECMO lasted 9 days. CDH repair was performed at 2.7 ± 2.3 days (median 1; range 1 – 12 days) after birth. In 3 cases, closure was progressive (6.9 and 12 days) and required a patch. Two children had concomitant preventive antireflux surgery. These data are reported in Table 1.

Twenty-one (65.6%) of the responders had current clinical problems related to CDH at the time of response. Digestive issues were found in 11 (34.3%) children, neurological issues in 3 (9.3%), respiratory issues in 7 (21.9%), orthopedic issues in 4 (12.5%) and psychological issues in 7 (21.9%) (Table 2). The SDQ was completed for 24 children. The mean SDQ dimension scores were in the range of the normal population or borderline. The responses are shown in Table 3.

**Table 2:** Current clinical problems of CHD survivors at the time of response

	**n (%)**
Digestive symptoms (total)	11 (34.4)
Vomiting	3 (9.3)
Gastro-oesophageal reflux	2 (6.3)
Constipation	3 (9.3)
Diarrhea	1 (3.1)
Abdominal pain	3 (9.3)
Medical treatment	3 (9.3)
Respiratory symptoms (total)	7 (21.9)
Dyspnea at rest	2 (6.3)
Exertional dyspnea	3 (9.3)
Nocturn dyspnea	1 (3.2)
Pain chest	0 (0)
Respiratory treatment	2 (6.3)
Repeated respiratory tract infection	4 (12.5)
Hospitalisation for respiratory trouble	3 (9.3)
Neuropsychologic symptoms (total)	8 (26.7)
Cerebral palsy	1 (3.1)
Mental retardation	1 (3.1)
Neurological following	1 (3.1)
Psychological following	7 (21.8)
Orthopedic symptoms (total)	4 (12.5)
Difficult walking	2 (6.3)
Kinesitherapy	2 (6.3)
Orthopedic treatment	1 (3.2)
Total	21 (65.6)

**Table 3:** Self-reported data from the CDH survivors and their parents

**1. Children**	**CHD**	**LEA**	**Norms**
Children’ QoL: Kidscreen-27 (0–100)*	**M ± SD**	**Diff (95% CI)**^**+**^	**Diff (95% CI)**^**++**^
Physical well-being	53.4 ± 10.7	5,9 (1,7 ; 10,1)	−11,6 (−15,6 ; -7,63)
Psychological well-being	53.4 ± 9.5	2,3 (−1,5 ; 6,3)	−23,8 (−27,2 ; -20,3)
Parents and autonomy	47.3 ± 7.9	−0, (−5,4 ; 3,5)	−14,3 (−17,4 ; -11,1)
Peers and social support	44.5 ± 9.5	0,1 (−5,0 ; 5,3)	−16,3 (−19,8 ; -12,8)
School	53.5 ± 10.0	3,0 (−1,2 ; 7,3)	−13,2 (−17,0 ; -9,44)
**Children’ behavioral : SDQ**	**M ± SD**		
Emotional symptoms	3.0 ± 2.7	-	-
Conduct problems	2.5 ± 1.7	-	-
Hyperactivity	4.1 ± 2.4	-	-
Peer problems	1.6 ± 2.4	-	-
Prosocial	8.2 ± 1.5	-	-
Total difficulties	11.3 ± 5.7	-	-
**2. Parents**			
Parents’ QoL: SF36 (0–100)*	**M ± SD**		
Physical function	92.5 ± 13.2	-	-
Social functioning	86.7 ± 19.1	-	-
Role physical	89.9 ± 15.3	-	-
Role emotional	63.2 ± 9.1	-	-
Mental health	71.4 ± 14.2	-	-
Vitality	71.7 ± 17.0	-	-
Body pain	83.8 ± 22.5	-	-
General health	79.7 ± 17.3	-	-
Physical composite score	55.9 ± 6.1	-	-
Mental composite score	46.7 ± 5.6	-	-

*M±SD* mean±standard deviation.

* Higher score, higher *QoL* level.

*LEA* long term leucemia survivors, norms, Kidscreen norms (sex- and gender- matched).

+ Diff (95% CI) *CHD* score – *LEA* score (95% confidence interval of difference); ++ Diff (95% CI), *CHD* score – norm score (95% confidence interval of difference).

### Quality of life of CDH survivors

**Figure 1:**
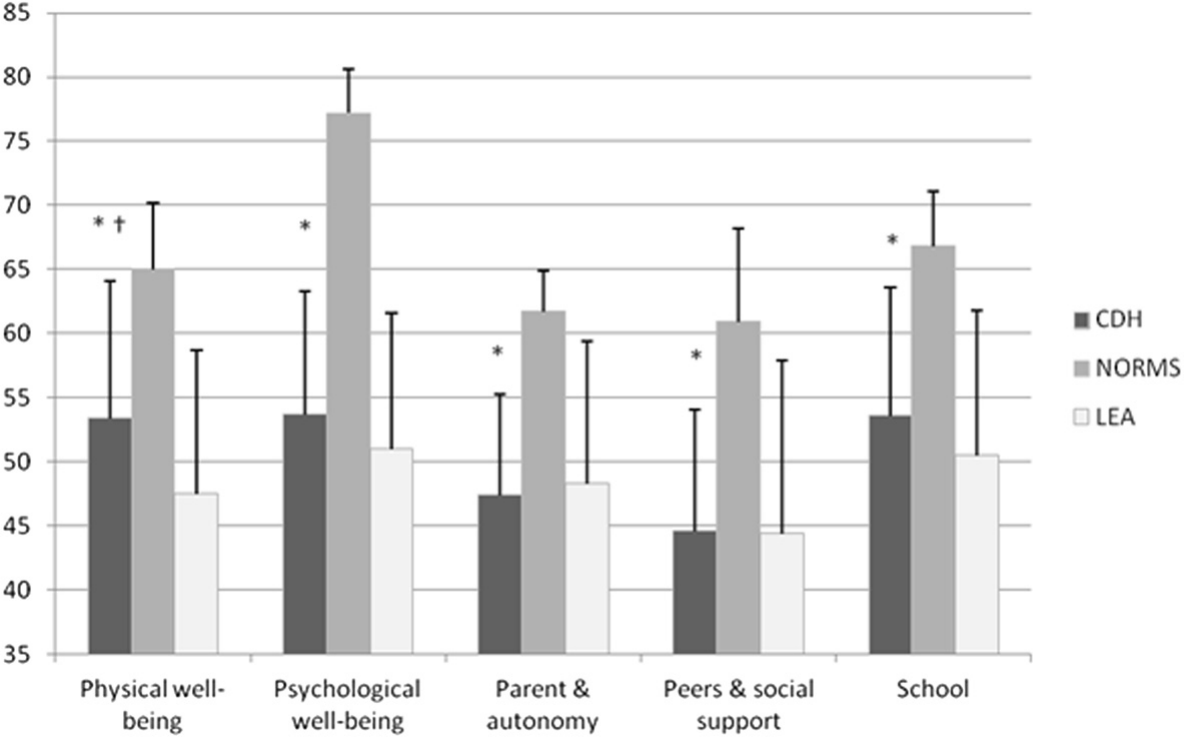
**Comparaison of Kidscreen 27 scores of CDH survivors with long term leucemia survivors (LEA), and norms.** * p < 0.001 between sample and Kidscreen norms (sex- and gender- matched). † p < 0.01 between sample and Kidscreen scores of LEA cohort.

The results of these scores are detailed in Table 3: the highest score was physical well-being, and the lowest score was the peers and social support score. Of the 5 dimensions of the Kidscreen-27, the scores of CDH survivors were significantly lower (by more than 12 points) compared with matched controls (Figure 1 and Table 3). Compared with 214 long-term survivors of childhood leukemia, the physical well-being dimension was significantly higher for CDH survivors, but there were no differences in the 4 other dimensions.

### Factors modulating the quality of life in CDH survivors

#### Modulation of children’s QoL

Children with GER at discharge had a significantly poorer score in 3 dimensions (psychological well-being, parents and autonomy, and peers and social support) of the Kidscreen-27. Antenatal diagnosis and length of stay in the ICU were also associated with lower scores in the Kidscreen-27 (psychological well-being and parents and autonomy for antenatal diagnosis and psychological well-being for length of ICU stay).

Neuropsychological issues in children were associated with a lower parents and autonomy score in the Kidscreen-27. Respiratory issues were associated with a lower peers and social support score.

Gender, age at the time of response, surfactant administration, high-frequency oscillation, nitric oxide use, vasopressor use, birth term, birth weight, mechanical ventilation duration, type of surgery, and orthopedic issues were not associated with differences in Kidscreen-27 scores.

These results are detailed in Table 4.

**Table 4:** Children’ quality of life (Kidscreen-27): association with socio-demographic parameters and initial management

		**PhWB**	**PsWB**	**ParAu**	**PSS**	**Sch**
Antenatal diagnosis*	**Yes**	50.0 ± 9.2	49.0 ± 6.6	45.0 ± 8.9	44.2 ± 10.6	51.0 ± 9.0
	**No**	58.8 ± 11.0	60.3 ± 9.6	50.5 ± 5.3	45.1 ± 7.8	57.3 ± 10.6
	**p**	0.056	**0.003**	**0.035**	0.613	0.114
GER at discharge*	**Yes**	49.9 ± 10.3	48.9 ± 7.5	43.7 ± 8.1	40.5 ± 10.0	50.7 ± 8.4
	**No**	56.2 ± 10.4	57.0 ± 9.7	50.7 ± 6.2	48.0 ± 7.9	55.8 ± 10.8
	**p**	0.095	**0.016**	**0.009**	**0.013**	0.213
Age**	**Coeff**	−0.057	−0.020	0.099	0.358	0.047
	**p**	0.762	0.914	0.639	0.062	0.807
ICU LOS**	**Coeff**	−0.249	−0.397	−0.062	0.374	−0.281
	**p**	0.184	**0.03**	0.772	0.055	0.140
Current respiratory problems*	**Yes**	48.3 ± 12.5	53.6 ± 10.5	42.2 ± 7.7	38.2 ± 7.5	56.6 ± 12.3
	**No**	54.85 ± 9.9	53.28 ± 9.5	49.02 ± 7.4	46.66 ± 9.3	52.78 ± 9.5
	**p**	0.068	0.924	0.074	**0.031**	0.334
Current digestive problems*	**Yes**	51.9 ± 9.3	51.2 ± 7.2	48.5 ± 7.9	45.9 ± 12.1	51.9 ± 5.9
	**No**	54.2 ± 11.5	54.5 ± 10.6	46.8 ± 8.09	43.7 ± 7.69	54.4 ± 11.6
	**p**	0.572	0.504	0.600	0.832	0.506
Current neuropsychological problems*	**Yes**	47.3 ± 8.9	51.5 ± 9.1	41.3 ± 6.6	39.8 ± 8.0	48.4 ± 9.2
	**No**	54.9 ± 10.3	54.2 ± 10.3	48.3 ± 6.8	44.9 ± 8.8	55.5 ± 10.3
	**p**	0.051	0.657	**0.042**	0.200	0.101

* mean±standard deviation, p p-value Mann–Whitney test.

** Spearman’s correlation coefficient *p*, p-value Spearman’s test.

*PhWB*, Physical well-being *PsWB*, Psychological well-being *Par&Au*, Parents and autonomy *PSS*, Peers and social support *Sch*, School.

higher score represents higher level of *QoL*.

Bold values, p < 0.05.

#### Parents’ quality of life

**Figure 2:**
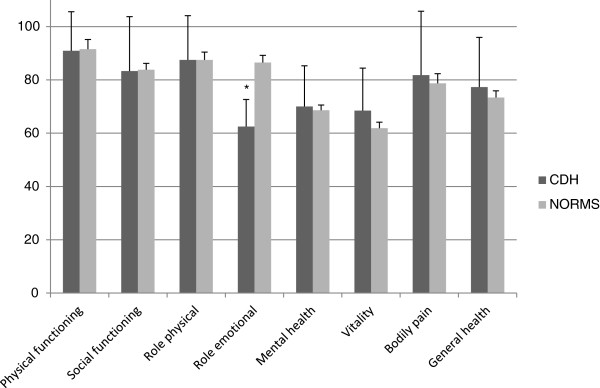
**Comparisons of SF36 dimensions scores between the parents of CDH survivors and norms.** * p < 0.05 between sample and SF-36 norms (sex- and gender- matched).

There were no differences between the parents of CDH survivors and matched controls in SF36 scores except for the role emotional dimension, which was significantly lower for parents of CDH survivors (Figure 2).

### Modulation of parents’ QoL

The mental component score in the SF36 of parents of CDH survivors was significantly altered in cases of right-sided CDH, antenatal diagnosis and GER at discharge. GER at discharge was also associated with a lower score in the general health and role physical dimensions. Difficult abdominal closure was associated with a lower bodily pain dimension score and physical component score, while a higher age of children at the moment of response was associated with a poorer physical functioning dimension score, and a longer ICU stay was associated with a lower role physical dimension score. The gender of the child, age at the moment of response, surfactant administration, high-frequency oscillation, nitric oxide use, vasopressor use, birth term, birth weight, and mechanical ventilation duration were not associated with differences in the SF36 score.

These results are detailed in Table 5.

**Table 5:** Parents’ quality of life (SF36): associations with socio-demographic parameters and initial management

		**PF**	**SF**	**RP**	**RE**	**MH**	**Vi**	**BP**	**GH**	**PCS**	**MCS**
Antenatal diagnosis*	**Yes**	91.5 ± 15.7	82.6 ± 22.3	87.5 ± 16.2	61.6 ± 10.4	68.5 ± 13.9	67.4 ± 16.3	80.4 ± 23.8	75.0 ± 18.9	55.0 ± 6.7	45.1 ± 5.7
	**No**	93.8 ± 9.2	92.3 ± 12.0	93.3 ± 13.8	65.4 ± 6.7	75.8 ± 14.1	78.1 ± 16.5	88.5 ± 20.6	86.8 ± 12.1	57.3 ± 5.1	49.0 ± 4.9
	**p**	0.683	0.301	0.185	0.508	0.134	0.072	0.245	0.085	0.290	**0.038**
Surgery*	**Simple**	92.8 ± 14.1	87.8 ± 19.2	91.8 ± 14.0	62.2 ± 8.6	71.1 ± 14.9	72.0 ± 18.0	87.1 ± 21.8	80.8 ± 16.0	56.9 ± 5.6	46.3 ± 5.9
	**Others**	91.0 ± 8.2	85.0 ± 20.5	80.0 ± 19.5	68.3 ± 10.9	73.0 ± 11.0	70.0 ± 12.0	66.4 ± 19.6	74.0 ± 24.1	51.1 ± 7.0	48.7 ± 4.3
	**p**	0.238	0.637	0.091	0.329	0.955	0.933	**0.014**	0.655	**0.048**	0.254
GER at discharge*	**Yes**	93,7 ± 3,2	83,0 ± 6,2	87,0 ± 3,7	60,7 ± 2,8	69,8 ± 3,6	66,9 ± 3,4	84,1 ± 6,7	70,8 ± 5,0	55,2 ± 1,6	45,0 ± 1,4
	**No**	91,5 ± 3,5	89,7 ± 3,6	92,3 ± 4,0	65,2 ± 1,8	72,8 ± 3,8	75,78 ± 4,9	83,5 ± 5,1	87,4 ± 3,0	56,5 ± 1,6	48,1 ± 1,4
	**p**	0,848	0,711	**0,029**	0,304	0,487	0,159	0,794	**0,008**	0,339	**0,042**
Age**	**Coeff**	−0.378	−0.174	0.212	−0.262	−0.204	−0.132	−0.142	−0.110	−0.147	−0.154
	**p**	**0.036**	0.348	0.252	0.155	0.281	0.488	0.446	0.564	0.437	0.417
ICU LOS**	**Coeff**	−0.019	−0.201	−0.417	−0.111	−0.023	−0.189	−0.291	−0.289	−0.350	−0.042
	**p**	0.919	0.286	**0.022**	0.558	0.904	0.325	0.118	0.128	0.063	0.829
Current respiratory problems*	**Yes**	87,0 ± 19,0	71,4 ± 25,7	87,5 ± 19,1	63,1 ± 6,6	72,9 ± 15,2	64,3 ± 13,8	68,1 ± 32,2	63,9 ± 24,8	50,8 ± 8,6	45,9 ± 5,0
	**No**	94,1 ± 11,0	91,1 ± 14,5	90,6 ± 14,4	63,2 ± 9,8	71,0 ± 14,2	73,9 ± 17,4	88,3 ± 17,2	84,5 ± 11,2	57,5 ± 4,3	46,9 ± 5,9
	**p**	0,068	**0,038**	0,980	0,958	0,637	0,399	0,147	**<0.001**	0,059	0,391
Current digestive problems*	**Yes**	90,4 ± 14,3	83,8 ± 22,0	90,0 ± 15,3	65,0 ± 9,5	65,8 ± 17,1	70,6 ± 15,6	78,7 ± 27,0	76,1 ± 15,8	55,1 ± 5,8	45,4 ± 6,7
	**No**	93,5 ± 12,8	88,1 ± 17,8	89,9 ± 15,6	62,3 ± 8,98	74,3 ± 11,9	72,2 ± 17,9	86,2 ± 20,3	81,5 ± 18,1	56,3 ± 6,35	47,3 ± 5,13
	**p**	0,248	0,642	0,787	0,727	0,174	0,824	0,342	0,320	0,356	0,509
Current neuropsychological problems*	**Yes**	96,3 ± 5,2	90,6 ± 18,6	90,6 ± 10,0	62,5 ± 14,1	70,3 ± 11,1	68,8 ± 14,2	88,6 ± 20,6	70,5 ± 18,7	56,2 ± 6,0	46,0 ± 5,7
	**No**	92,7 ± 13,3	86,3 ± 19,7	91,1 ± 15,1	63,9 ± 6,6	72,5 ± 15,3	74,1 ± 18,2	80,4 ± 23,8	83,5 ± 16,6	56,0 ± 6,5	47,3 ± 5,7
	**p**	0,751	0,396	0,348	0,914	0,367	0,400	0,384	0,055	0,959	0,476

* mean±standard deviation *p*, p-value Mann–Whitney test.

** Spearman’s correlation coefficient *p*, p-value Spearman’s test.

*PF* physical functioning, *SF* social functioning, *RP* role physical, *RE* role-emotional, *MH* mental health, *VI* vitality, *BP* bodily pain, *GH* general health, *PCS* physical composite score *MCS*, mental composite score, higher score represents higher level of *QoL*, Bold values, p < 0.0.

## Discussion

The most important finding of this study is that CDH survivors have an altered QoL compared with controls and a similar QoL compared with children with another pathology associated with long-term consequences (childhood leukemia survivors). As expected, the physical dimension is altered in CDH survivors, but the score remains higher than the score of childhood leukemia survivors, who are faced to a severe disease and known to have severe physical sequelae [23].

Nearly two thirds of CDH survivors have clinical problems. The most frequently reported problems are gastrointestinal issues. Similar data had already been reported in previous studies. We show here that these issues severely impact QoL.

Behavioral issues can be an important determinant of QoL. We explored this problem using the SDQ. Recent data have proposed SDQ norms for French children [25]. All mean sub-scores in our population were in the normal range. However, this questionnaire did not test mild issues, such as attention and concentration deficit reported by Peetsold *et al.*[15].

At discharge, CDH survivors have a good vital prognosis but are not cured and require attentive evaluation and management of long-term morbidity to limit QoL alteration. Our data suggest a lower QoL than 2 previous studies [12,14]. Several reasons can explain these differences. One explanation could be the sample characteristics. In Peetsold’s study, the authors used the Child Health Questionnaire (CHQ) to explore the QoL of CDH child survivors aged 6 to 16 years [15]. The authors showed that the level of QoL did not differ from norms, except for general health perception and physical functioning in the CHQ-PF50 (completed by parents of children younger than 10 years) and general health perception in the CHQ-CF87 (completed by children 10 years old and older). The children in that study were born between 1987 and 1999. Therapeutic management has been progressively modified during this period, and it is possible that some children did not receive all the treatments that are currently available. For example, the first clinical report of INO use was published in 1992 [26]. Therefore, survivors were probably the less serious cases, had less sequelae and higher quality of life in childhood. In our study, we chose the period after 1999 to ensure that the most recent treatments were used. The second published study compared the QoL of 111 CDH survivors with the QoL of 286 survivors of congenital anorectal malformation and a control group [13]. Fifty-nine CDH child survivors between 1 and 16 years were included. The authors found that both physical and psychological/emotional dimensions were altered. In that study, all the patients were born before 1996, meaning that most of the children were not treated according to most recent recommendations. Furthermore, the results were expressed by different age groups, limiting data analysis.

A second explanation of the discrepancies between our results and previous findings may rely on the modalities of QoL assessment. In the two previous studies, QoL was assessed using well-validated questionnaires, but the content of these questionnaires relied on either the literature or experts to determine the domains and concerns that are important for the individuals, although it is now generally accepted that the content of QoL measures should be directly derived from affected individuals [27]. The questionnaire used in our study, the Kidscreen questionnaire, is based on both literature and focus groups (children, parents and workers in the field). The choice of the QoL questionnaires can also be discussed. Poley *et al.*[13] used the Taiqol questionnaire, which has two different non-mergeable versions according to age, to evaluate the QoL, restricting the significance of the results. The Kidscreen allows for merging child and adolescent scores.

The important results of our study are the potential determinants of QoL, which were poorly explored in previous studies, although the small sample size prevented multivariate analysis. Several parameters were linked to QoL in univariate analysis and could help clinicians to identify children with a high risk of having altered QoL early. The first parameter was prenatal diagnosis, which was associated with lower QoL. An explanation of this result is that prenatally undiagnosed CDH is associated with lower severity. Pathophysiology of CDH is complex and not yet completely elucidated, however a “dual hit” pathogenesis [28] is widely admitted. Abnormal development, early in the pregnancy causes diaphragmatic defect and development troubles affecting both lungs. Secondly, compression by bowel herniation causes hypoplasia. We hypothesis to explain our findings, that when the hernia occurs late in pregnancy the diagnosis is not made because CDH occurs at the end of gestation after ultrasound control in the third trimester. In these cases, pulmonary consequences caused by the “second hit” could be lower, decreasing impact lung functioning at birth [29].

Gastroesophageal reflux is a major complication of CDH and one of the most reported complaints of patients [30]. The presence of GER at discharge is associated with a lower score in 3 dimensions of the Kidscreen-27 and 3 dimensions in the SF36 in parents. As widely reported in the literature, GER is frequent in this population and requires early diagnosis and treatment. Interestingly, digestive issues, including GER, at the time of the response were not linked with lower children’s QoL. GER at discharge most likely reflects the severity of disease and may be associated with several problems responsible for issues impacting QoL later.

The ICU length of stay was associated with a lower QoL in CDH survivors. The hospital length of stay had been found as the only factor associated with lower QoL by Peetsold *et al.*[15]. These results are concordant and confirm that QoL alteration occurs for the most severe patients. Furthermore, excepted the disease and its sequelae, congenital malformation [31] and ICU hospitalization [32] at birth can be responsible by themselves for parental attachment troubles with late consequences in parents/child relation and possible impact on QoL.

Unlike others authors who found a lower QoL in younger children, we did not find any correlation between age and Kidscreen score. A possible explanation is that the QoL of CDH survivors improves after 10 or 12 years, and we did not explore this older population in our study, while other authors included children up to 16 years.

We found few differences between the QoL of parents of CDH survivors and controls; however, the emotional dimension score was lower in parents of CDH survivors, which is in agreement with a previous study in which the authors explored the QoL of the family and parents of 53 CDH survivors [14] using the Child Health Ratings Inventories General Health Module Parent Report. This questionnaire does not explore all domains of QoL; for example, it neglects social aspects. In that study, the children were born between 1991 and 1999 during the modification period of therapeutic management of CDH. We chose to use the SF36 questionnaire, which is the most widely used questionnaire to evaluate QoL in adults. However, the parenthood status of the norms was not available. It is well-know that the process of becoming/being parent or being childless may influence the QoL [33,34]. Future studies should specifically study the parenting influence. For the first time to our knowledge, the QoL of CDH survivors and the QoL of their parents were simultaneously measured.

Several limitations of our study must be mentioned. The sample size did not allow for a multivariate approach accounting for potential confounding factors, and moderate associations were possibly missed due to low power. This limitation is a frequent problem in this area. Nevertheless we can assume that a majority of individuals with CHD living in the geographic area had access to the participant centers. Replication of these findings in larger groups of patients is required. For the same reason, ECMO was not analyzed because only one child in this study was treated by ECMO. ECMO as rescue can lead to specific morbidity and could significantly impact the QoL of CDH survivors. Another important problem is that some data have been retrospectively collected, which may lead to bias. For example, we could not evaluate and compare the severity of patients because admission severity scores were poorly reported on charts and different between participating PICUs. Furthermore, although it appears that pregnancy termination for CDH is rare in our prenatal medical center, we have no reliable data to confirm this hypothesis. Therefore, the high mortality rate observed cannot be interpreted as poor results of treatment. Similarly, initial ultrasound and MRI results were not available for enough patients to be included in univariate analysis. A third limitation is that the questionnaires were sent by mail, and we did not meet the children or their family. This increases the missing data rate and decreases the reliability of responses to clinical data. We considered that hearing and vision problems, which are frequently reported in CDH survivors, were too subjective to be evaluated by a postal questionnaire. Future research should be provided medical data based on clinical assessments.

In conclusion, this is the first study providing data on CDH child survivors treated according to the most recent practices, and for the first time, the QoL of children and their parents was studied simultaneously. The impact of CDH on QoL seems to be important and must be understood by clinicians who treat these children and their parents. Prenatal diagnosis and GER at discharge are two markers of QoL alteration and must be considered by clinicians. Finally, all existing studies include a small number of patients, limiting the interpretation of results. It is important to perform multicentric studies to better evaluate the QoL of these patients and its determinants.

## Abbreviations

CDH: Congenital diaphragmatic hernia; QoL: Quality of life; PICU: Paediatric intensive care unit; INO: Inhaled nitric oxide; ECMO: Extracorporeal membrane oxygenation; GER: Gastroesophageal reflux; SF36: Short form 36 questionnaire; SDQ: Strengths and difficulties questionnaire; TPV: Total pulmonary volume; EPV: Expected pulmonary volume.

## Competing interests

The authors declare that they have no competing financial interests and no competing non-financial interests.

## Authors’ contributions

FM: Conception and design of study, interpretation of data, drafting the manuscript. KB: Conception and design of the study, statistical analysis and interpretation of data, drafting the manuscript. AG: Acquisition of data, interpretation of data, drafting the manuscript. PLC: Conception and design of study, interpretation of results and validation of the manuscript. TM: Participating to design of study, interpretation of results and final approval of the submitted manuscript. SH: Conception and design of study, participating to acquisition of data, revising the manuscript KC: Acquisition of imaging data, interpretation of data, revising the manuscript. JB: Acquisition of data, interpretation of data, revising the manuscript. CM: Conception and design of the study, supervision of research and final approval of the manuscript. PA: conception and design of the study, interpretation of data, final approval of the submitted manuscript. All authors read and approved the final manuscript.
